# Repeat-dose toxicity of human umbilical cord mesenchymal stem cells via subcutaneous injection in NOG mice

**DOI:** 10.3389/fcell.2025.1558310

**Published:** 2025-03-03

**Authors:** Lijuan Xia, Jinjin Shao, Qian Yang, Chengda Zhang, Zhiqi Xie, Linying Wang, Cong Xu, Siming Zhang, Jing Liu, Fang Liu, Yuhua Shi, Liqiang Gu, Xiaobo Lin, Jiahong Wang, Ying Chen, Yunxiang Chen, Xin Pan, Feifei Wu, Ruolang Pan, Jinfeng Liang, Lijiang Zhang

**Affiliations:** ^1^ Key Laboratory of Drug Safety Evaluation and Research of Zhejiang Province, Center of Safety Evaluation and Research, Hangzhou Medical College, Hangzhou, China; ^2^ Wuyi First People’s Hospital, Affiliated Hospital, School of Medicine, Hangzhou City University, Hangzhou, China; ^3^ Qingshan Lake Science and Technology Innovation Center, Hangzhou Medical College, Hangzhou, China; ^4^ Zhejiang Key Laboratory of Cell-Based Drug and Applied Technology Development, S-Evans Biosciences Co., Ltd., Hangzhou, China; ^5^ Zhejiang Center for Drugs and Cosmetics Evaluation, Zhejiang Province Food and Drug Administration, Hangzhou, China

**Keywords:** human umbilical cord mesenchymal stem cells, subcutaneous injection, toxicity assessment, NOG mice, foamy cells

## Abstract

**Background:**

Stem cell therapy shows promise for treating skin diseases and enhancing medical aesthetics. However, safety data for subcutaneous injection of stem cells remain limited. In this study, we evaluated the toxicity of human umbilical cord mesenchymal stem cells (hUC-MSCs) in NOD. Cg-Prkdc^scid^IL2rg^tm1Sug^/JicCrl (NOG) mice.

**Methods:**

Mice received subcutaneous hUC-MSC injections at doses of 2.5 × 10^7^ and 2.0 × 10^8^ cells/kg on days 1, 8, 12, 16, and 20, followed by withdrawal and observation for 6 weeks. Toxicity was assessed through clinical observation, behavioral analysis, pathology, organ weight measurements, and histopathology. hUC-MSC distribution was determined via validated quantitative (q)PCR and colonization was assessed using immunohistochemistry.

**Results:**

No abnormal effects on clinical responses, body weight, or food intake were observed following five repeated hUC-MSCs administrations, except for masses at the administration site in the high-dose group. Mouse activity levels increased in both dose groups 6 h post-final injection. Foamy cells were observed under the pleural membrane in high-dose mice. hUC-MSCs primarily colonized and were distributed within skin tissues 24 h after the last administration.

**Conclusion:**

The no-observed-adverse-effect level for subcutaneous hUC-MSC administration in NOG mice over 3 weeks was 2.5 × 10^7^ cells/kg. Our results will help in advancing the clinical use of hUC-MSCs, particularly for treating conditions such as atopic dermatitis.

## 1 Introduction

The skin, the largest organ of the human body, acts as the primary physiological defense (Eckhart and Zeeuwen, 2018). It performs essential functions, including secretion, excretion, metabolism, absorption, temperature regulation, and sensation (Roosterman et al., 2006). Impairment of these functions can result in various skin diseases, (Bäsler et al., 2016), including difficult-to-treat conditions such as sensitive skin, psoriasis, steroid-induced dermatoses, and acne. Traditional treatments often address symptoms rather than underlying causes, leading to frequent relapses. Advances in regenerative medicine have introduced stem cell therapy, offering new hope for treating skin diseases (Hoang et al., 2022). Currently, stem cells have shown significant potential in various related fields, including pemphigus, systemic sclerosis, systemic lupus erythematosus, psoriasis, vitiligo, wound healing, hair loss, and medical aesthetics (Anderi et al., 2018; Farabi et al., 2024; Chang et al., 2021; Kaffenberger et al., 2013; Yuan et al., 2019; Gregory et al., 2024; Wang et al., 2017; Li et al., 2019).

Human umbilical cord mesenchymal stem cells (hUC-MSCs), derived from Wharton’s jelly of the umbilical cord, offer several advantages, including ease of collection, high purity, abundant availability, strong activity; in addition, their differentiation capabilities are comparable to those of embryonic stem cells (Mousaei et al., 2022; Ding et al., 2015). These characteristics make them a valuable resource among mesenchymal stem cell sources. Additionally, hUC-MSCs have benefits such as the absence of ethical concerns, immune rejections, and harm to donors and recipients (Xie et al., 2020). Published animal and clinical trials demonstrate the therapeutic potential of hUC-MSCs for skin conditions, including wound healing, skin aging, psoriasis, atopic dermatitis, and scleroderma (Hua et al., 2023; Ren et al., 2023; Zhang et al., 2024; Liu et al., 2024; Liang et al., 2018). Subcutaneous injection is a common method for administering treatments in dermatology and aesthetic medicine (Yang et al., 2024; Pan et al., 2023). However, research on the adverse reactions associated with subcutaneous injection of stem cells is limited.

In this repeat-dose study, severely immunodeficient NOD/Shi-scid/IL-2 Rγ null (NOG) mice received subcutaneous injections of hUC-MSCs for 3 weeks (four doses), followed by a 6-week recovery period, and we assessed the general toxicities, including adverse reactions, potential target organs for toxicity, effects on the central nervous system, distribution and colonization, and the no-observed-adverse-effect level (NOAEL). This study provides valuable preclinical data for clinical research and highlights indicators requiring close monitoring in clinical practice.

## 2 Materials and methods

### 2.1 Cells

The hUC-MSCs and diluents used in this study were sourced from S-Evans Biosciences in Hangzhou and derived from healthy umbilical cord tissues, consistent with previous studies (Pan et al., 2023; Shao et al., 2023). The tissues were minced for primary culture and cells were treated with 0.25% trypsin-ethylenediaminetetraacetic acid (EDTA) at 80%–90% confluence for passage. Passage five hUC-MSCs were utilized in this study. Flow cytometry confirmed the expression of CD105, CD73, and CD90 (≥95%) and the absence of CD14, CD19, CD34, CD45, and HLA-DR (≤2%), which aligned with hUC-MSC characteristics. Adipogenic and osteogenic differentiation assays were performed to identify hUC-MSC phenotypes. The trypan blue exclusion assay revealed that 95% of the cells were viable. The cells were prepared on the day of use and kept on ice, with transplantation performed within 6 h of preparation.

### 2.2 Animals

NOG mice (6–7 weeks old, half male and half female) were purchased from Beijing Vital River Laboratory Animal Technology Company (China). The mice were housed in individually ventilated cages under controlled conditions: 22.5°C–24.4°C, 23%–69% relative humidity, central air ventilation ≥15 times/h, and 12 h light/dark cycles. Cage ventilation frequency was set at 30–80 times/h. The facilities used in this study were accredited by the Association for Assessment and Accreditation of Laboratory Animal Care (AAALAC, #001489). All experimental animals were bred and used for scientific research purposes, with procedures reviewed and approved by the Institutional Animal Care and Use Committee of the Safety Evaluation Research Center of Hangzhou Medical College (approval number: GLP-2022-116).

### 2.3 Experimental design

The study was part of the preclinical safety evaluation of the “Human Umbilical Cord Mesenchymal Stem Cell Injection” for the treatment of moderate to severe atopic dermatitis. The study design was developed in accordance with the International Council for Harmonization of Technical Requirements for Pharmaceuticals for Human Use (ICH) guidelines M3 (R2) and related technical guidelines from China’s National Medical Products Administration (NMPA, 2013; ICH, 2009; NMPA, 2017).

**FIGURE 1:**
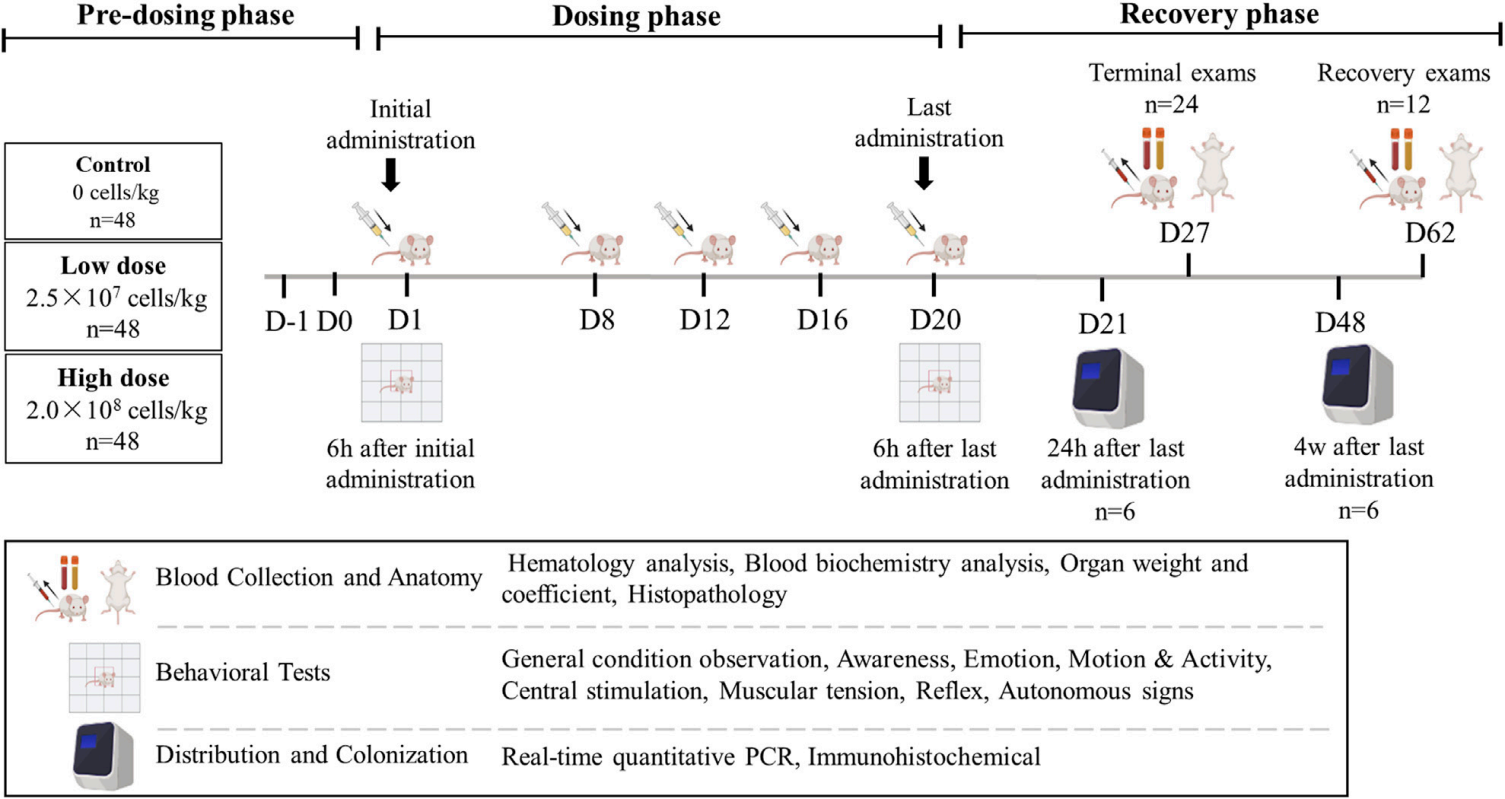
Overview of the study design.

A total of 144 mice (half male and half female) were randomly assigned to groups using a weight-balanced randomization method. Each group (control, low-dose, and high-dose) contained 48 mice. The mice were subcutaneously injected with cell diluent (Multiple Electrolytes Injection; Baxter, United States) containing hUC-MSCs at doses of 2.5 × 10^7^ cells/kg (effective dose for treating atopic dermatitis) or 2.0 × 10^8^ cells/kg (maximum feasible dose in mice) on days 1, 8, 12, 16, and 20. hUC-MSCs were injected subcutaneously into the same site each time, with an injection volume of 20 mL/kg for both the low and high dose groups. The injection site was consistent across all injections to maintain uniformity in the experimental design. Of the 48 mice in each group, 24 (half male and half female) were dissected for analysis on day 27 (1 week after the last treatment), and 12 (half male and half female) were dissected on day 62 (6 weeks after the last treatment, at the end of the recovery period). The remaining 12 mice in each group were used for behavioral and tissue distribution experiments and did not undergo clinical examination. Six mice (half male and half female) were dissected on day 21 for tissue distribution and immunohistochemistry, while six more (half male and half female) were dissected on day 48 for tissue distribution and colonization (Figure 1).

### 2.4 Clinical examination

Throughout the trial, daily records were kept on various aspects of the subjects, including physical appearance, injection sites, behavior patterns, skin condition, respiratory function, and the state of their mouths and nostrils. Observations were also made on glandular secretions, as well as the characteristics of feces and urine, and any instances of mortality. Body weight was tracked twice weekly. During the dosing phase, food consumption was monitored twice weekly, and once weekly for the remainder of the trial. Two-stage planned dissections were performed throughout the trial: terminal examinations on day 27 and recovery examinations on day 62. Hematological (including coagulation) and chemical (including electrolyte) examinations were performed, along with histopathological assessments, gross dissections, and the weighing of major organs.

### 2.5 Histopathological analysis

For tissue sampling, mice were fasted for ≥12 h with free access to water and anesthetized via intraperitoneal injection of sodium pentobarbital (100 mg/kg). Following a comprehensive external examination, blood was collected from the abdominal vena cava, after which the animals were euthanized for systematic necropsy. All major tissues and organs were inspected, and gross pathological findings were documented.

Ocular tissues were preserved in Davidson’s fixative, while other tissues were fixed in 10% neutral buffered formalin for 24–48 h. Fixed tissues were processed through graded ethanol series, embedded in paraffin, and sectioned at 3 μm thickness. Tissue sections were stained with hematoxylin and eosin (H&E) for microscopic evaluation by a board-certified pathologist.

### 2.6 Behavioral evaluation

Mice underwent behavioral assessments 6 h after the initial and final injections using a functional observation battery (FOB) test (Gauvin et al., 2016). This standardized protocol allowed for a detailed assessment of various behavioral and neurological functions, including cage observation, handheld visual observation, open-field observation, handheld assisted observation, and other factors (Table 1).

**TABLE 1:** The behavioral evaluation via FOB test in NOG mice 6 h after last administration of hUC-MSCs.

Parameter			Control	Low dose	High dose
General condition observation	Feeding	Normal	12/12	12/12	12/12
	Drinking	Normal	12/12	12/12	12/12
	Vertical hair	NA	12/12	12/12	12/12
	Abnormal posture	NA	12/12	12/12	12/12
	Abnormal behavior	NA	12/12	12/12	12/12
	Grooming behavior	Normal	12/12	12/12	12/12
Awareness	Awaken	Normal	12/12	12/12	12/12
	The difficulty of removal	1	1/12	0/12	0/12
		2	11/12	12/12	12/12
	Head contact	Normal	12/12	12/12	12/12
	Visual positioning reflex	Normal	12/12	12/12	12/12
	Passive response	Normal	12/12	12/12	12/12
Emotion	Abnormal vocalization and crowing	NA	12/12	12/12	12/12
	Aggressiveness towards cage-mates	NA	12/12	12/12	12/12
	Restless and restless	NA	12/12	12/12	12/12
	Hairing (Times/2 min)		4 ± 2	2 ± 1**	3 ± 2*
Motion&Activity	Position	Normal	12/12	12/12	12/12
	Autonomous activities	Normal	12/12	12/12	12/12
	Total length of the route (mm)		1,554 ± 732	3,316 ± 1,897**	2,958 ± 1,459*
	The length of the route in the central area (mm)		324 ± 264	431 ± 416	381 ± 376
	Average speed inside the box (mm/s)		13 ± 6	28 ± 16**	25 ± 12*
	Standing (times/2 min)		2 ± 2	6 ± 5**	4 ± 3
	Ataxic gait	NA	12/12	12/12	12/12
Central stimulation	Convulsions, tremors, convulsions	NA	12/12	12/12	12/12
	Auditory startle reflex	Normal	12/12	12/12	12/12
	Pinch tail pain reflex	Normal	12/12	12/12	12/12
Muscular tension	Hypotonic gait	NA	12/12	12/12	12/12
	Abdominal tension	Normal	12/12	12/12	12/12
	Limb tension	Normal	12/12	12/12	12/12
	Grip	Normal	12/12	12/12	12/12
Reflex	Ocular reflex	Normal	12/12	12/12	12/12
	Auricular reflex	Normal	12/12	12/12	12/12
	Planar righting reflection	Normal	12/12	12/12	12/12
	Spatial righting reflection	Normal	12/12	12/12	12/12
Autonomous signs	Micturition	Normal	12/12	12/12	12/12
	Defecation	Normal	12/12	12/12	12/12
	Shed tears	NA	12/12	12/12	12/12
	Drooling	NA	12/12	12/12	12/12
	Pupillary response	Normal	12/12	12/12	12/12
Others	Body temperature (℃)		37.2 ± 0.6	37.8 ± 0.8	37.8 ± 0.3*
	Death	NA	12/12	12/12	12/12

Note: Data are presented as the number of occurrences/total number of animals, or mean ± SD. “*” or “**” indicate statistically significant differences at *p* < 0.05 and *p* < 0.01, respectively, when compared to the control group. “NA” indicates that the behavior did not occur.

### 2.7 Distribution and colonization analysis

After the final injections, six mice from both the low- and high-dosage groups and two from the control group were euthanized at 24 h (day 1) and 4 weeks (day 28) post-injection. Blood (with EDTA anticoagulant) and organs, including lungs, heart, liver, spleen, kidneys, brain, spinal cord, adipose tissue, gonads, and uterus, were harvested from each mouse, with approximately 30 mg of tissue taken from each. Samples were kept on ice before being stored at temperatures below −70°C for tissue distribution studies.

Quantitative (q)PCR was used to analyze human *SRY* gene DNA in mouse tissues to determine the distribution of hUC-MSCs *in vivo* (Ye et al., 2023). The SRY gene, located on the Y chromosome, is a specific marker for male cells and is used here to track the distribution of hUC-MSCs transplanted into NOG mice. The primer sequences used were SRY-F (5ʹ-TGT​CGC​ACT​CTC​CTT​GTT​TTT-3ʹ) and SRY-R (5ʹ-TGG​GTC​GCT​TCA​CTC​TAT​CCT-3ʹ). The parameters were set as follows: 50°C for 2 min, 95°C for 2 min, 95°C for 15 s, 64°C for 1 min (40 cycles), 95°C for 15 s, 60°C for 1 min, and 95°C for 15 s. A standard curve was established by linear regression of SRY plasmid Ct and concentration log values to determine the human SRY DNA content in plasma samples. Tissues that tested positive for SRY by PCR were subjected to immunohistochemical analysis for human mitochondrial protein expression to confirm the presence of hUC-MSCs.

The biodistribution of hUC-MSCs in NOG mouse tissues was quantified using a validated immunohistochemical (IHC) approach targeting human-specific mitochondrial protein (Ye et al., 2023). Tissue sections were processed using the HRP-DAB detection system with the following antibody protocol: primary incubation with anti-mitochondria antibody (1:200 dilution, Cat# GR3307132-27, Abcam) followed by secondary detection with horseradish peroxidase (HRP)-conjugated goat anti-mouse IgG (1:1,000 dilution, Cat# GR3405228-11, Abcam).

### 2.8 Hematological and biochemical analyses

For hematological evaluation, approximately 0.3 mL of blood was collected from the abdominal vena cava into K₂EDTA anticoagulant tubes and analyzed within 4 h at ambient temperature (20°C–25°C). For coagulation testing, an additional 0.3 mL blood sample was transferred to sodium citrate anticoagulant tubes (3.2% concentration), followed by centrifugation at 3,000 × g for 10 min at 25°C to obtain plasma. Coagulation analyses were completed within 4 h at ambient temperature or within 8 h when stored at 2°C–8°C. Biochemical analysis involved collecting approximately 0.6 mL of blood into plain tubes without anticoagulants. Samples were clotted in a 37°C water bath for 30 min, then centrifuged at 3,000 × g for 10 min to isolate serum. Serum samples were maintained at 2°C–8°C and subjected to biochemical testing within 48 h. The main reagents, test items, and methods are detailed in the Supplementary Tables S6–S8.

### 2.9 Statistical analysis

SPSS Statistics (v.23.0, IBM, United States) was used for statistical analyses. Measurement data, including body weight, food intake, hematological indicators, serum biochemical indicators, and organ weight and coefficient, are presented as the mean ± standard deviation (SD). One-way analysis of variance (ANOVA) was performed for significance testing. When a significant difference was found between the overall groups, pairwise comparisons were conducted. Levene’s test was used to assess the homogeneity of variance. If groups were homoscedastic, the least significant difference method was used; if heteroscedastic, the Games–Howell test was used for pairwise comparisons. Count data, such as behavioral and pathological incidences, were described as fractions, while non-parametric tests (such as the Kruskal–Wallis test) were employed for significance analysis. If a statistically significant difference was found, pairwise group comparisons were performed. A *p-*value of < 0.05 was considered statistically significant.

## 3 Results

### 3.1 General observations, body weight, and food consumption

**FIGURE 2:**
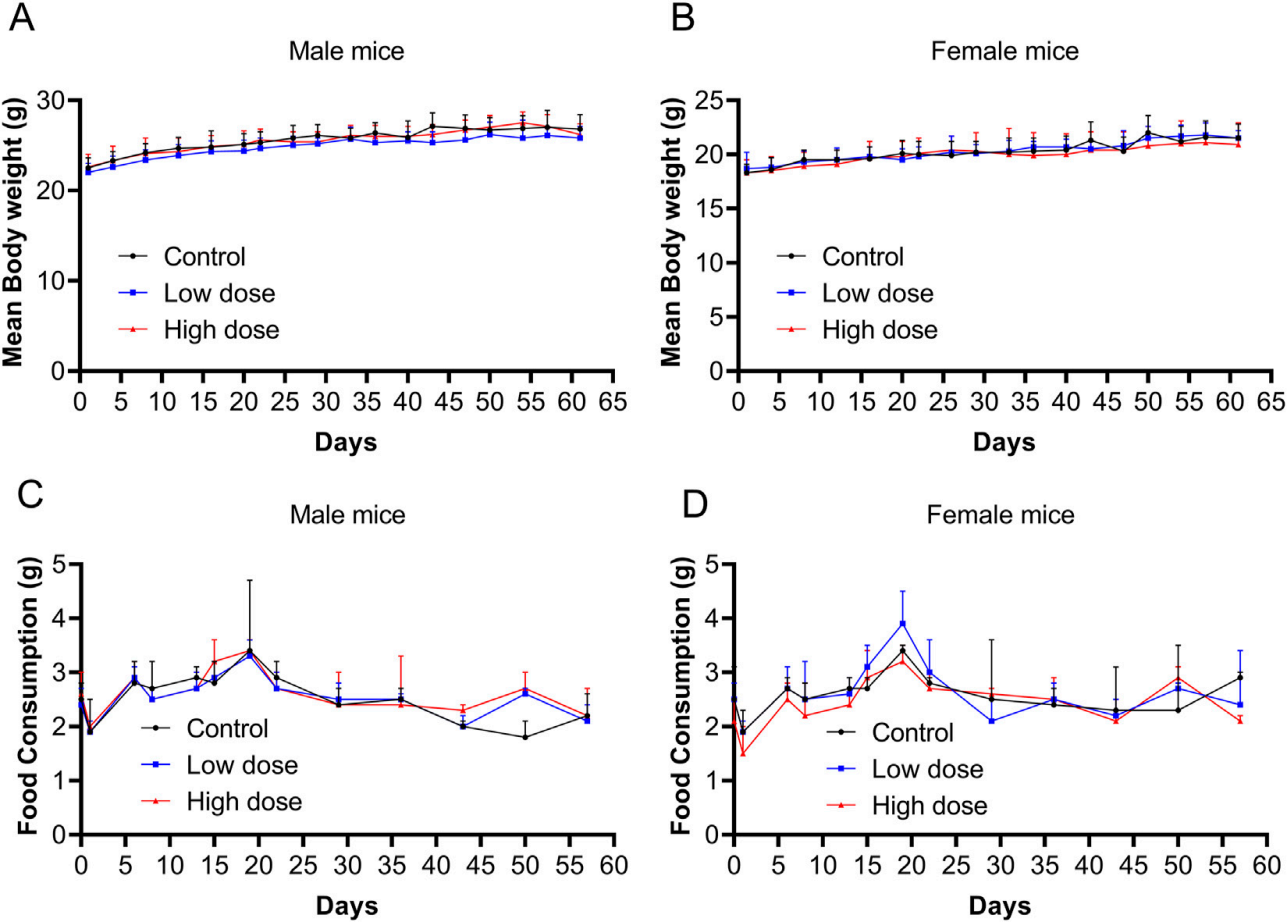
Body weight and food consumption after repeated doses of human umbilical cord mesenchymal stem cells (hUC-MSCs). Body weight of **(A)** male and **(B)** female mice from days 0–62. Food consumption of **(C)** male and **(D)** female mice from days 0–57. Data are expressed as the mean ± standard deviation and were analyzed using one-way analysis of variance (ANOVA).

Throughout the experimental period, no significant abnormalities were observed in the low- and high-dose groups, and no mouse mortality occurred, which was similar to the vehicle control group. All groups (vehicle control, low-dose, and high-dose) exhibited brief and reversible swelling at the administration site immediately after each dose. Starting on day 2, mice in the high-dose group began to develop soft skin lumps at the administration site, which persisted throughout the recovery period. No significant differences were observed in body weight or food consumption between the low- and high-dose groups and the control group (Figure 2).

### 3.2 Behavioral evaluation via FOB test

No abnormalities were observed in the behavioral evaluation indicators for the low- and high-dose groups after the first administration (Supplementary Table S1). At 6 h after the final administration, motor activity indicators (total distance traveled in the box, average speed in the box, number of standing episodes in the box) increased in the low- and high-dose groups. However, other general observations, including consciousness, mood, central excitation, muscle tone, reflexes, and autonomic signs, showed no significant abnormalities (Table 1).

### 3.3 Hematology and biochemical findings

At the end of the drug withdrawal and recovery periods, no abnormalities in hematological indicators related to hUC-MSCs were observed in the low- and high-dose groups (Table 2; Supplementary Table S2). During the drug withdrawal examination, no abnormalities in biochemical indicators were found in these groups related to hUC-MSCs (Supplementary Table S3). However, during the recovery period examination, a slight increase in total protein and albumin was observed in the low- and high-dose groups. This increase was not present during the drug withdrawal examination (Table 3). The magnitude of these fluctuations was minimal, with no clear dose-related trend, and they were considered to be incidental variations without significant toxicological implications.

**TABLE 2:** Hematology analysis of NOG mice treated with hUC-MSCs in the examination of drug withdrawal.

Parameter	Control	Low dose	High dose
Number of animals	12	12	12
WBC (10^3^/μL)	0.26 ± 0.10	0.45 ± 0.36	0.33 ± 0.15
#NEUT (10^3^/μL)	0.19 ± 0.08	0.37 ± 0.34	0.23 ± 0.11
#LYMPH (10^3^/μL)	0.02 ± 0.01	0.03 ± 0.02	0.03 ± 0.02
#MONO (10^3^/μL)	0.01 ± 0.01	0.01 ± 0.01	0.01 ± 0.01
#EOS (10^3^/μL)	0.04 ± 0.03	0.05 ± 0.04	0.05 ± 0.06
#BASO (10^3^/μL)	0.00 ± 0.00	0.00 ± 0.00	0.00 ± 0.00
#LUC (10^3^/μL)	0.000 ± 0.00	0.001 ± 0.003	0.001 ± 0.003
%NEUT (%)	74.9 ± 8.0	75.8 ± 14.9	71.2 ± 11.3
%LYMPH (%)	8.2 ± 3.3	7.9 ± 5.1	10.1 ± 4.1
%MONO (%)	2.6 ± 1.9	2.0 ± 1.8	2.6 ± 1.8
%EOS (%)	13.6 ± 6.0	13.4 ± 15.3	15.0 ± 13.5
%BASO (%)	0.4 ± 0.6	0.3 ± 0.5	0.3 ± 0.2
%LUC (%)	0.2 ± 0.3	0.6 ± 0.5	0.8 ± 0.8
RBC (10^6^/μL)	8.32 ± 0.17	8.71 ± 0.41*	8.19 ± 0.26
HGB (g/dL)	131 ± 5	136 ± 9	128 ± 4
HCT (%)	45.1 ± 1.0	47.3 ± 3.0	44.5 ± 1.6
MCV (fL)	54.3 ± 0.7	54.3 ± 1.2	54.3 ± 0.8
MCH (Pg)	15.7 ± 0.6	15.6 ± 0.5	15.7 ± 0.3
MCHC (g/L)	289 ± 10	288 ± 5	289 ± 3
RDW (%)	13.5 ± 0.4	13.6 ± 0.4	13.7 ± 0.3
PLT (10^3^/μL)	1,346 ± 296	1,557 ± 335	1,553 ± 344
MPV (fL)	9.9 ± 0.9	10.3 ± 0.6	9.9 ± 0.4
%RETIC (%)	3.56 ± 0.51	3.70 ± 0.42	3.78 ± 0.32
#RETIC (10^9^/L)	295.3 ± 40.5	321.7 ± 37.5	309.5 ± 25.3
PT (s)	8.4 ± 1.2	7.8 ± 0.6	7.9 ± 0.8
Fbg (g/L)	1.769 ± 0.435	1.876 ± 0.346	1.498 ± 0.279
APTT (s)	30.5 ± 10.1	23.6 ± 2.8	28.6 ± 7.8

Note: Data are expressed as mean ± SD. “*” indicates a statistically significant difference at *p* < 0.05 when than the control group; Abbreviations: WBC, white blood cell; RBC, red blood cell count; HGB, hemoglobin concentration; HCT, hematocrit; MCV, mean cell volume; MCH, mean cell hemoglobin; MCHC, mean cell hemoglobin concentration; RDW, red blood cell distribution width; PLT, platelets; MPV, mean platelet volume; NEUT, neutrophiles; LYMPH, lymphocytes; MONO, monocytes; EOS, eosinophils; BASO, basophiles; LUC, large unstained cells; RETIC, reticulocyte; PT, prothrombin time; Fbg, fibrinogen; APTT, activated partial thromboplastin time.

**TABLE 3:** Blood biochemistry analysis of NOG mice treated with hUC-MSCs in the examination at the end of the recovery period.

Parameter	Control	Low dose	High dose
Number of animals	6	6	6
ALT (IU/L)	24.73 ± 2.81	22.69 ± 0.73	23.74 ± 2.22
AST (IU/L)	57.56 ± 12.90	68.23 ± 8.65	81.55 ± 23.29
ALP (IU/L)	67.42 ± 10.92	66.48 ± 20.27	71.00 ± 10.53
T.BIL (umol/L)	3.163 ± 1.556	3.033 ± 0.524	2.716 ± 1.354
CK (IU/L)	77.73 ± 34.23	265.38 ± 245.77	460.48 ± 277.81
T.P (g/L)	41.02 ± 1.34	44.17 ± 2.10*	44.48 ± 2.60*
ALB (g/L)	26.81 ± 0.96	28.82 ± 0.90*	28.91 ± 1.59**
GLO (g/L)	14.21 ± 0.98	15.35 ± 1.56	15.57 ± 1.54
A/G	1.90 ± 0.15	1.89 ± 0.18	1.87 ± 0.18
GLU (mmol/L)	7.449 ± 0.315	7.615 ± 0.899	6.801 ± 0.304
BUN (mmol/L)	10.015 ± 0.510	10.247 ± 0.355	9.589 ± 1.009
Crea (umol/L)	31.33 ± 0.81	34.97 ± 3.15	33.91 ± 1.05**
T.CHO (mmol/L)	1.563 ± 0.110	1.690 ± 0.243	1.682 ± 0.270
TG (mmol/L)	0.844 ± 0.156	0.800 ± 0.147	0.811 ± 0.196
K^+^ (mmol/L)	4.11 ± 0.24	4.10 ± 0.42	4.45 ± 0.35
Na^+^ (mmol/L)	153.3 ± 1.8	153.5 ± 0.5	152.8 ± 1.3
Cl^−^ (mmol/L)	125.8 ± 4.8	122.2 ± 2.7	120.3 ± 1.3
Ca (mmol/L)	1.98 ± 0.05	2.07 ± 0.07	2.05 ± 0.09

Note: Data are expressed as mean ± SD. “*” or “**” indicates a statistically significant difference at *p* < 0.05 and *p* < 0.01, respectively, than the control group.

Abbreviation: ALT, alanine aminotransferase; AST, aspartate aminotransferase; TP, total protein; ALB, albumin; TBIL, total bilirubin; ALP, alkaline phosphatase; r-GT, r-Glutamyltransferase; GLU, glucose; BUN, blood urea nitrogen; Crea, Creatinine; CHO, cholesterol; TG, triglyceride; CK, creatine phosphokinase; GLO, globulin; A/G, albumin/globulin ratio.

### 3.4 Histopathology

At the end of the drug withdrawal and recovery period, no abnormalities related to hUC-MSCs were observed in the weights and coefficients of various organs in the low- and high-dose groups (Table 4; Supplementary Table S4). Gross examination results showed that at the end of the drug withdrawal and recovery period, all mice in the high-dose group had subcutaneous transparent/white elevations at the administration site, and no macroscopic pathological changes related to the test article were observed in the remaining tissues and organs of mice in all groups during both periods.

**TABLE 4:** Organ weight and coefficient of NOG mice treated with hUC-MSCs in the examination of drug withdrawal.

Parameter		Male mice			Female mice		
		Control	Low dose	High dose	Control	Low dose	High dose
Number of animals		12	12	12	12	12	12
Body weight (g)		23.8 ± 1.1	23.0 ± 1.3	24.1 ± 1.3	18.6 ± 1.1	18.6 ± 1.2	19.4 ± 1.1
Brain	Weight (g)	0.496 ± 0.030	0.493 ± 0.021	0.483 ± 0.024	0.491 ± 0.024	0.501 ± 0.023	0.483 ± 0.019
	Organ coefficient (%)	2.087 ± 0.103	2.147 ± 0.112	2.008 ± 0.122	2.646 ± 0.172	2.698 ± 0.219	2.499 ± 0.105*
Heart	Weight (g)	0.115 ± 0.013	0.112 ± 0.015	0.118 ± 0.017	0.095 ± 0.009	0.097 ± 0.006	0.100 ± 0.014
	Organ coefficient (%)	0.484 ± 0.053	0.488 ± 0.057	0.490 ± 0.062	0.509 ± 0.046	0.521 ± 0.032	0.515 ± 0.074
	Organ brain coefficient	0.232 ± 0.024	0.227 ± 0.027	0.244 ± 0.028	0.193 ± 0.017	0.194 ± 0.010	0.206 ± 0.025
Liver	Weight (g)	0.933 ± 0.070	0.885 ± 0.076	0.967 ± 0.110	0.696 ± 0.062	0.715 ± 0.081	0.741 ± 0.062
	Organ coefficient (%)	3.921 ± 0.237	3.843 ± 0.187	4.005 ± 0.341	3.738 ± 0.200	3.835 ± 0.337	3.830 ± 0.209
	Organ brain coefficient	1.881 ± 0.110	1.795 ± 0.143	2.003 ± 0.232	1.418 ± 0.115	1.434 ± 0.208	1.537 ± 0.128
Spleen	Weight (g)	0.019 ± 0.003	0.020 ± 0.004	0.026 ± 0.005**	0.021 ± 0.005	0.024 ± 0.007	0.026 ± 0.007
	Organ coefficient (%)	0.078 ± 0.011	0.088 ± 0.015	0.107 ± 0.017**	0.113 ± 0.025	0.132 ± 0.040	0.132 ± 0.034
	Organ brain coefficient	0.038 ± 0.005	0.041 ± 0.009	0.054 ± 0.011**	0.043 ± 0.009	0.049 ± 0.017	0.053 ± 0.014
Kidney	Weight (g)	0.342 ± 0.033	0.325 ± 0.026	0.342 ± 0.029	0.227 ± 0.022	0.227 ± 0.018	0.233 ± 0.014
	Organ coefficient (%)	1.436 ± 0.093	1.411 ± 0.076	1.415 ± 0.072	1.221 ± 0.076	1.217 ± 0.068	1.205 ± 0.047
	Organ brain coefficient	0.689 ± 0.042	0.658 ± 0.040	0.707 ± 0.063	0.463 ± 0.042	0.453 ± 0.033	0.483 ± 0.025
Adrenal gland	Weight (g)	0.006 ± 0.002	0.006 ± 0.003	0.006 ± 0.002	0.006 ± 0.001	0.007 ± 0.002	0.006 ± 0.001
	Organ coefficient (%)	0.026 ± 0.009	0.026 ± 0.011	0.025 ± 0.009	0.034 ± 0.005	0.040 ± 0.010	0.033 ± 0.007
	Organ brain coefficient	0.012 ± 0.004	0.012 ± 0.005	0.012 ± 0.005	0.013 ± 0.002	0.015 ± 0.003	0.013 ± 0.003
Testis/Uterus	Weight (g)	0.180 ± 0.020	0.182 ± 0.014	0.176 ± 0.016	0.120 ± 0.040	0.123 ± 0.055	0.112 ± 0.044
	Organ coefficient (%)	0.758 ± 0.078	0.793 ± 0.070	0.734 ± 0.079	0.651 ± 0.232	0.674 ± 0.332	0.577 ± 0.231
	Organ brain coefficient	0.364 ± 0.042	0.370 ± 0.032	0.365 ± 0.034	0.247 ± 0.085	0.247 ± 0.107	0.231 ± 0.092
Epididymis/Ovary	Weight (g)	0.080 ± 0.008	0.077 ± 0.009	0.080 ± 0.009	0.023 ± 0.007	0.021 ± 0.003	0.023 ± 0.003
	Organ coefficient (%)	0.336 ± 0.028	0.334 ± 0.039	0.332 ± 0.041	0.126 ± 0.039	0.113 ± 0.019	0.117 ± 0.014
	Organ brain coefficient	0.161 ± 0.016	0.156 ± 0.015	0.166 ± 0.023	0.048 ± 0.014	0.042 ± 0.007	0.047 ± 0.006

Note: Data are expressed as mean ± SD. “*” or “**” indicates a statistically significant difference at *p* < 0.05 and *p* < 0.01 when compared to the control group; Organ coefficient (%) = g organ weight/g body weight × 100%; Organ brain coefficient g organ weight/g brain weight.

**FIGURE 3:**
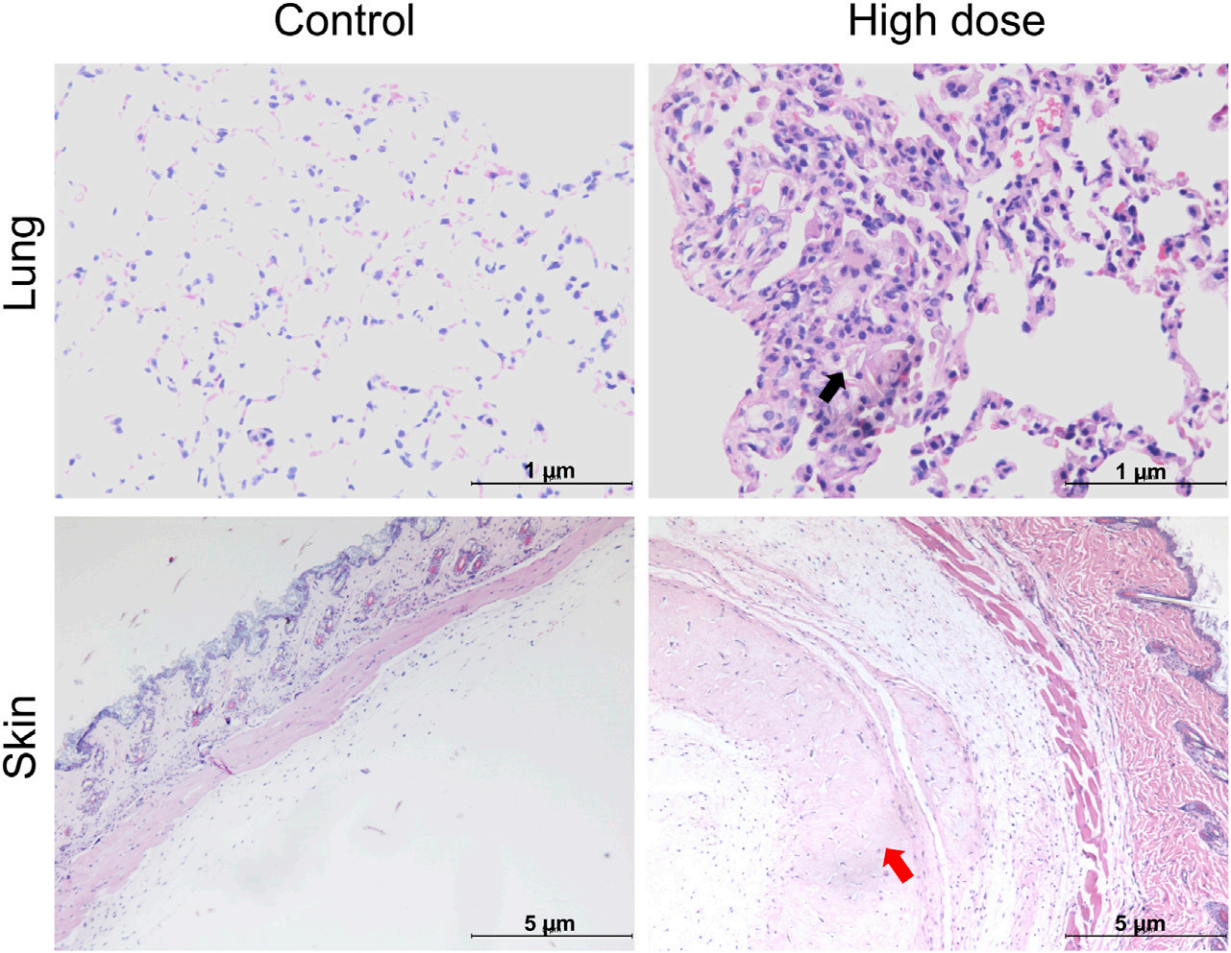
Histopathological images of the lung and skin of NOD. Cg-Prkdc^scid^IL2rg^tm1Sug^/JicCrl (NOG) mice after repeated administration of human umbilical cord mesenchymal stem cells (hUC-MSCs). Black arrow denotes foamy cells under the pleural membrane of the lung; red arrow indicates cellular mass under the skin.

Histopathological examination results showed that at the examination of drug withdrawal, some mice in the low-dose group (3♂2♀/24 mice) and all mice in the high-dose group (12♂12♀/24 mice) had visible cell clusters at the administration site, which were dose-related. A few animals in the control group and the low- and high-dose groups (4♀2♂/24 mice, 5♀1♂/24 mice, and 7♀5♂/24 mice, respectively) had mild to slight subpleural foamy cell aggregation in the lungs, with increasing trends in incidence and severity in the high-dose group compared with those in the control group. During the examination of the recovery period, there was a trend of recovery for the cell clusters at the administration site, and no obvious abnormalities were observed in the lungs of the high-dose group. No abnormal pathological changes related to the test article were observed in the remaining tissues and organs of mice in all groups during both periods (Figure 3; Table 5).

**TABLE 5:** Main lesion incidence and grading of NOG mice treated with hUC-MSCs.

Tissues/lesion	Group	Drug withdrawal						At the end of the recovery period					
		Control		Low dose		High dose		Control		Low dose		High dose	
	Gender	♀	♂	♀	♂	♀	♂	♀	♂	♀	♂	♀	♂
	Total number of animals	12	12	12	12	12	12	6	6	6	6	6	6
Lung Subcapsular foam cell aggregation	Degree of lesion: 2	0	1/12	0	0	0	1/12	—	—	—	—	—	—
	Degree of lesion: 1	2/12	3/12	1/12	5/12	5/12	6/12	1/6	0	2/6	2/6	0	0
	Number of occurrences	2/12	4/12	1/12	5/12	5/12	7/12	1/6	0	2/6	2/6	0	0
Administration site Cell clusters	Degree of lesion: 3	0	0	0	0	12/12	12/12	—	—	—	—	—	—
	Degree of lesion: 2	0	0	0	0	0	0	0	0	0	0	3/6	5/6
	Degree of lesion: 1	0	0	3/12	2/12	0	0	0	0	1/6	1/6	3/6	1/6
	Number of occurrences	0	0	3/12	2/12	12/12	12/12	0	0	1/6	1/6	6/6	6/6
Adrenal gland Subcapsular hyperplasia	Degree of lesion: 2	1/12	3/12	NA	NA	0	3/12	1/6	2/6	NA	NA	0	0
	Degree of lesion: 1	0	3/12	NA	NA	0	0	0	2/6	NA	NA	0	4/6
	Number of occurrences	1/12	6/12	NA	NA	0	3/12	1/6	4/6	NA	NA	0	4/6
Epididymis Vacuolar degeneration of epididymal head	Degree of lesion: 2	1/12	NA	NA	NA	0	NA	—	—	—	—	—	—
	Degree of lesion: 1	5/12	NA	NA	NA	4/12	NA	1/6	NA	NA	NA	1/6	NA
	Number of occurrences	6/12	NA	NA	NA	4/12	NA	1/6	NA	NA	NA	1/6	NA

Note: “NA” indicates that such an item does not exist. The degree of the lesion is “1” for mild, “2” for mild, “3” for moderate, “4” for severe, and “5” for severe.

### 3.5 Distribution and colonization

**FIGURE 4:**
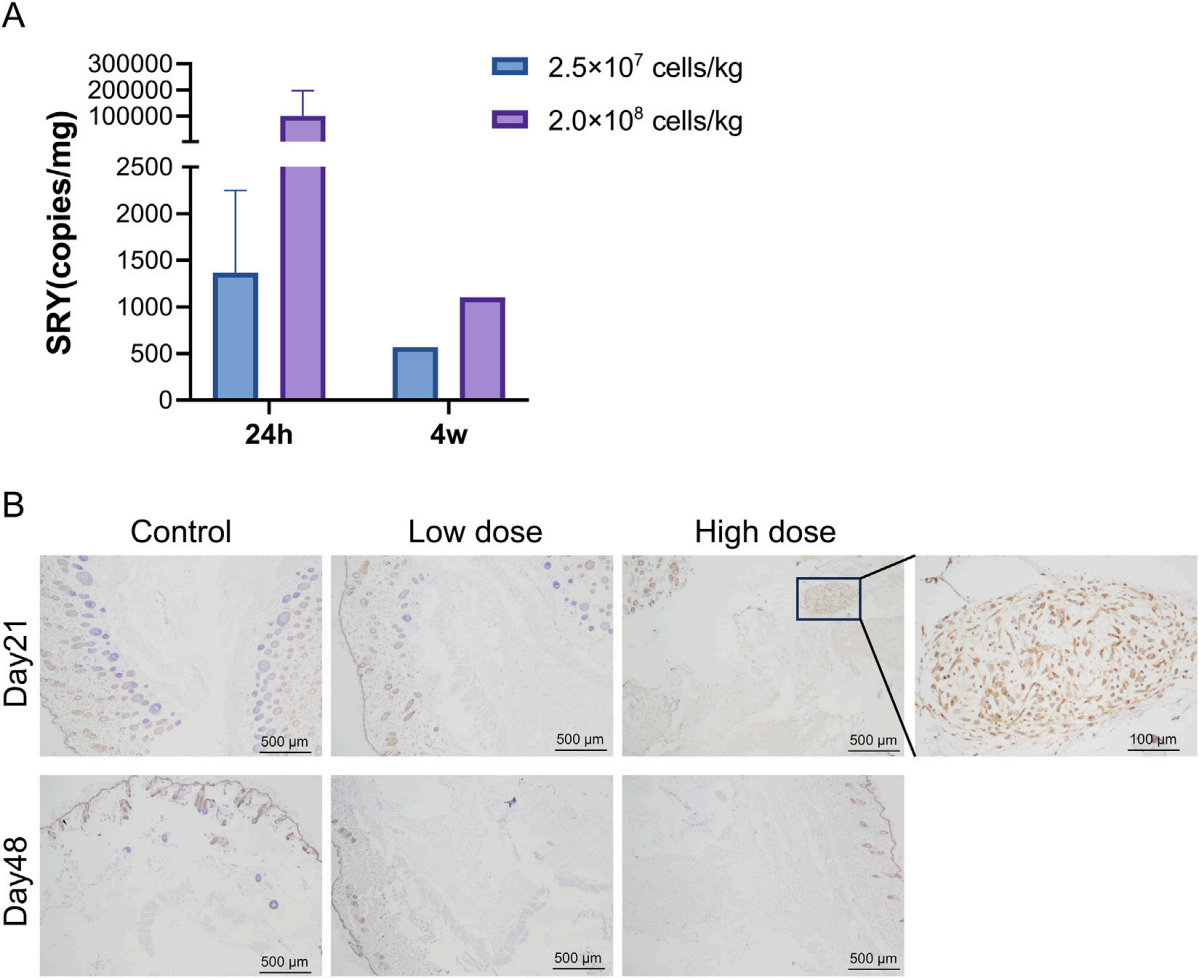
Distribution of human umbilical cord mesenchymal stem cells (hUC-MSCs). In the skin of NOD. Cg-Prkdc^scid^IL2rg^tm1Sug^/JicCrl (NOG) mice. **(A)** Concentration of human *SRY* DNA in the skin of NOG mice treated with hUC-MSCs (copies/mg, *n* = 6). **(B)** Immunohistochemistry (IHC) for mitochondria of skin tissues. Scale bar: 100 μm. Black arrowheads indicate positive regions. Day 21 = 24 h after the last administration; Day 48 = 4 weeks after the last administration.

qPCR results showed that hUC-MSCs were mainly distributed in the skin tissue at the administration site, with average contents of 1.37 × 10^3^ and 1.02 × 10^5^ copies/mg for the low- and high-dose groups, respectively, 24 h after the last administration. After 4 weeks of recovery following drug withdrawal, hUC-MSCs remained in the skin tissue of the administration site in one male mouse from each group (1♂/6 mice), with concentrations of 5.69 × 10^2^ and 1.10 × 10^3^ copies/mg in the low- and high-dose groups, respectively. No hUC-MSCs were found in other tissues during both periods (Figure 4A). Although individual samples from the lungs (1♀/6 mice in the low-dose group), kidneys (1♂/6 mice in the high-dose group), and muscles (1♀/6 mice in the high-dose group) showed measurable values, no apparent consistency or dose-dependency was found. Immunohistochemical analysis of colonization confirmed that the uterus was negative for hUC-MSCs (Supplementary Table S5). Thus, the measured values in these samples were considered false positives.

Immunohistochemical analysis revealed that 24 h after the last administration, hUC-MSCs were found in the skin tissue at the administration site in only three out of six mice in the high-dose group. The remaining mice in the high-dose group and all mice in the low-dose group showed no observable hUC-MSCs at the administration site. After 4 weeks of recovery following drug withdrawal, no hUC-MSCs were observed in the skin tissue at the administration site in mice from both the low- and high-dose groups, which is consistent with the PCR results (Figure 4B).

## 4 Discussion

In the clinical use of stem cell therapy for diseases, choosing the appropriate dosage form and route of administration is key to ensuring therapeutic effects and reducing potential side effects. The most common routes of administration for stem cells are injection, (Mahmoud et al., 2019; Yeung et al., 2022; Cerri et al., 2015), including arterial injection, intravenous injection, and local injection (subcutaneous, intramuscular, and intra-articular injections). Thrombotic events are the main adverse reactions to stem cell vascular injections (Shiratsuki et al., 2015). In our previous study, after NOG mice were administered hUC-MSCs via tail vein injection for 3 weeks (consecutive weeks with a total of six administrations), a significant pulmonary embolism occurred in the high-dose group at 3.5 × 10^7^ cells/kg, leading to reduced mouse activity and even death after the final administration (Shao et al., 2023). Local injection can directly inject stem cells into the lesion site or tissue requiring repair, reducing systemic distribution and the risk of embolism. This also ensures the concentration of stem cells in the target area, enhancing therapeutic effects (Zheng et al., 2024). Subcutaneous injection is a commonly used route of administration for skin-related diseases and aesthetic medicine (Li et al., 2024). Our results show that the NOAEL for subcutaneous injection of hUC-MSCs in NOG mice over 3 weeks is 2.5 × 10^7^ cells/kg, which is nearly 10 times higher than the 3.5 × 10^6^ cells/kg for intravenous injection. Moreover, in the high-dose group of 2.0 × 10^8^ cells/kg, no significant toxic side effects were observed except for the presence of subcutaneous nodules. These results suggest that local injection of stem cells has advantages in terms of safety, but current research on the safety of local stem cell injection is relatively limited. Therefore, additional studies are needed in the future to determine the safety, efficacy, and optimal dosing regimen of subcutaneous hUC-MSC injection to further promote its application in the treatment of skin diseases and dermatological aesthetic medicine.

The tissue distribution of stem cells is crucial for their therapeutic efficacy and safety. A study (Jian-liang and Deng-shun, 2009) demonstrated that after subcutaneously injecting 1 × 10^7^ bone marrow mesenchymal stem cells (BM-MSCs) at multiple sites in C57BL/6J mice for 8 weeks, LacZ staining revealed that BM-MSCs could enter capillaries and migrate to various organs through the bloodstream, including the lungs, brain, heart, liver, and renal cortex. No lumps were observed at the injection site, with only a few scattered cells present. The observed differences in cell distribution between our study and previous studies using BM-MSCs in C57 mice can be attributed to several factors. Firstly, the sources of the cells differed; we used hUC-MSCs, which may have distinct homing and migration properties compared with those of mouse-derived BM-MSCs. Secondly, the injection method varied; our study involved single-site subcutaneous injection, which may limit initial cell dispersion, whereas multiple-site injections in previous study facilitated more widespread distribution. Additionally, the intrinsic biological properties of hUC-MSCs, such as adhesion molecules and chemotactic responses, may differ from those of BM-MSCs, affecting migration patterns. These factors collectively explain the localized distribution of hUC-MSCs observed in our study compared with the systemic distribution reported in previous studies using BM-MSCs. These results indicate that the different outcomes of subcutaneous injections in different animal models may be influenced by factors such as the stem cells' characteristics, injection methods, dosing cycles and frequency, and animal species. Conversely, our research found that in NOG mice, hUC-MSCs administered subcutaneously for 3 weeks were mainly distributed in the skin at the injection site, leading to the formation of local lumps. H&E staining revealed that these lumps were cell aggregates rather than teratomas. The aggregates were most prevalent and largest in the high-dose group, indicating a dose-dependent relationship. Over time, these aggregates decreased in size, suggesting a trend towards recovery. Our findings are consistent with previous studies showing that hUC-MSCs do not form tumors or promote tumor growth (He et al., 2022). Instead, the observed cell aggregates were likely a result of the large-dose injection procedure. This finding is consistent with our earlier research on crab-eating macaques (Pan et al., 2023), where subcutaneous injection of 3 × 10^7^ cells/kg of umbilical cord mesenchymal stem cells twice a week for 3 weeks resulted in the formation of subcutaneous nodules and granulomatous lesions at the injection site. Behavioral assessments revealed a transient increase in locomotor activity 6 h post-final administration. Considering the general observations and gross pathological examination results, the increase in motor activity observed after the last administration is potentially related to discomfort caused by subcutaneous masses resulting from the injection of high concentrations of hUC-MSCs. Further research is crucial for guiding clinical applications.

Foamy cells are typically formed when monocytes and macrophages engulf excessive lipid substances, including cholesterol esters, and are commonly observed in pathological processes such as atherosclerosis (He et al., 2024). However, the aggregation of foamy cells in the lungs is rare and may indicate an inflammatory response or metabolic abnormality; however, the underlying mechanisms and impact on lung function remain unclear. Although our experimental results showed a trend of foamy cell aggregation under the pleura of mice in the high-dose group, we cannot conclusively attribute this aggregation to the administration of the test article owing to the lack of a significant difference among the groups, its occurrence in all groups, its mild severity, and its resolution after the recovery period. Future studies should explore the biological mechanisms of foamy cell aggregation, its influencing factors, and potential relationships with stem cells. Additionally, clinical monitoring of patient responses is essential to ensure the safety and efficacy of the treatment.

In our study, NOG mice were administered hUC-MSCs via subcutaneous injection over a period of 3 weeks, followed by a 6-week recovery phase. At a dose of 2.0 × 10^8^ cells/kg, mice developed localized skin lumps at the injection site. Both the 2.5 × 10^7^ and 2.0 × 10^8^ cells/kg dose groups exhibited increased activity levels in behavioral evaluations 6 h after the final dose. Slight elevations in total protein and albumin were noted at the end of the recovery period. However, upon comprehensive assessment, these behavioral and biochemical changes were deemed not to have significant toxicological relevance. Therefore, considering the absence of significant adverse effects at the lower dose, the NOAEL of subcutaneously administered hUC-MSCs in NOG mice for 3 weeks was 2.5 × 10^7^ cells/kg, with the primary distribution observed in the skin 24 h after administration. Subsequently, various comprehensive safety studies on hUC-MSCs were conducted, thereby supporting the application for a new drug clinical trial (IND) of “Human Umbilical Cord Mesenchymal Stem Cell Injection,” which was granted implied permission by the China National Drug Administration (acceptance number: CXSL2300670). The indication for this treatment is moderate to severe atopic dermatitis.

## Data Availability

The original contributions presented in the study are included in the article/Supplementary Material, further inquiries can be directed to the corresponding authors.
