# Supplementary material for: Repeat-dose toxicity of human umbilical cord mesenchymal stem cells via subcutaneous injection in NOG mice
Source: Front Cell Dev Biol. 2025 Mar 3;13:1558310. doi: 10.3389/fcell.2025.1558310 (PMC11911471; doi:10.3389/fcell.2025.1558310)
Supplement: Supplementary file 1 [file Table1.pdf]

Supplementary table 1. The behavioral evaluation via FOB test in NOG mice 6 hours after initial administration of hUC-MSCs.

Parameter			Control	Low dose	High dose
General condition observation	Feeding	Normal	12/12	12/12	12/12
	Drinking	Normal	12/12	12/12	12/12
	Vertical hair	NA	12/12	12/12	12/12
	Abnormal posture	NA	12/12	12/12	12/12
	Abnormal behavior	NA	12/12	12/12	12/12
	Grooming behavior	Normal	12/12	12/12	12/12
Awareness	Awaken	Normal	12/12	12/12	12/12
	The difficulty of removal	0	0/12	0/12	1/12
		1	7/12	1/12	0/12
		2	5/12	11/12	11/12
	Head contact	Normal	12/12	12/12	12/12
	Visual positioning reflex	Normal	12/12	12/12	12/12
	Passive response	Normal	12/12	12/12	12/12
Emotion	Abnormal vocalization and crowing	NA	12/12	12/12	12/12
	Aggressiveness towards cage-mates	NA	12/12	12/12	12/12
	Restless and restless	NA	12/12	12/12	12/12
	Hairing (Times/2min)		1 ± 2	1 ± 1	1 ± 1
Motion & Activity	Position	Normal	12/12	12/12	12/12
	Autonomous activities	Normal	12/12	12/12	12/12
	Total length of the route (mm)		5698 ± 1904	4565 ± 2065	6133 ± 2293
	The length of the route in the central area (mm)		989 ± 474	756 ± 497	1129 ± 413
	Average speed inside the box (mm/s)		47 ± 16	38 ± 17	51 ± 19
	Standing (times/2 minutes)		8 ± 5	5 ± 4	9 ± 5
	Ataxic gait	NA	12/12	12/12	12/12
Central stimulation	Convulsions, tremors, convulsions	NA	12/12	12/12	12/12
	Auditory startle reflex	Normal	12/12	12/12	12/12
	Pinch tail pain reflex	Normal	12/12	12/12	12/12
Muscular tension	Hypotonic gait	NA	12/12	12/12	12/12
	Abdominal tension	Normal	12/12	12/12	12/12
	Limb tension	Normal	12/12	12/12	12/12
	Grip	Normal	12/12	12/12	12/12
Reflex	Ocular reflex	Normal	12/12	12/12	12/12
	Auricular reflex	Normal	12/12	12/12	12/12
	Planar righting reflection	Normal	12/12	12/12	12/12
	Spatial righting reflection	Normal	12/12	12/12	12/12
Autonomous signs	Micturition	Normal	12/12	12/12	12/12
	Defecation	Normal	12/12	12/12	12/12
	Shed tears	NA	12/12	12/12	12/12
	Drooling	NA	12/12	12/12	12/12
	Pupillary response	Normal	12/12	12/12	12/12
Others	Body temperature (°C)		37.9 ± 0.6	37.7 ± 0.6	38.1 ± 0.3
	Death	NA	12/12	12/12	12/12

Note: The data were expressed as number of occurrences/total number of animals, or mean ± SD. "NA" indicates that this behavior was not appeared.

Supplementary table 2. The hematology analysis of NOG mice treated with hUC-MSCs in the examination at the end of the recovery period.

Parameter	Control	Low dose	High dose
Number of animals	6	6	6
WBC (10^3/μL)	0.82± 0.44	0.94± 0.74	0.63± 0.17
#NEUT (10^3/μL)	0.61± 0.35	0.79± 0.68	0.45± 0.15
#LYMPH (10^3/μL)	0.12± 0.04	0.08± 0.03	0.10± 0.02
#MONO (10^3/μL)	0.05± 0.05	0.02± 0.01	0.04± 0.01
#EOS (10^3/μL)	0.04± 0.01	0.05± 0.03	0.04± 0.01
#BASO (10^3/μL)	0.00± 0.00	0.00± 0.00	0.00± 0.00
#LUC (10^3/μL)	0.002± 0.004	0.000± 0.000	0.003± 0.005
%NEUT (%)	73.9± 6.0	79.2± 7.7	70.7± 6.0
%LYMPH (%)	15.8± 5.4	10.9± 5.0	16.3± 4.2
%MONO (%)	4.9± 2.3	2.8± 1.3	5.5± 1.8
%EOS (%)	4.9± 1.6	6.6± 2.5	6.5± 2.7
%BASO (%)	0.2± 0.3	0.2± 0.3	0.4± 0.2
%LUC (%)	0.2± 0.3	0.4± 0.3	0.7± 0.8
RBC (10^6/μL)	8.26± 0.13	8.27± 0.21	8.32± 0.25
HGB (g/dL)	129± 3	127± 6	127± 4
HCT (%)	45.1± 1.0	44.3± 1.5	43.9± 1.2
MCV (fL)	54.6± 0.7	53.6± 0.5*	52.8± 0.8**
MCH (Pg)	15.6± 0.3	15.4± 0.4	15.3± 0.3
MCHC (g/L)	286± 3	288± 5	290± 3
RDW (%)	13.1± 0.3	12.9± 0.1	12.8± 0.2
PLT (10^3/μL)	1562± 241	1562± 133	1774± 251
MPV (fL)	7.5± 0.3	7.9± 0.3	8.0± 0.3
%RETIC (%)	2.86± 0.60	2.79± 0.27	2.14± 0.32*
#RETIC (10^9/L)	235.9± 48.0	230.4± 23.6	177.7± 26.6
PT (s)	7.1± 0.3	7.2± 0.5	7.2± 0.3
Fbg (g/L)	2.260± 0.802	2.308± 0.278	2.492± 0.705
APTT (s)	28.3± 13.2	20.9± 5.7	21.9± 0.4

Note: The data were expressed as mean ± SD. "*"or"**" indicates a statistically significant difference at p <0.05 and p <0.01 when compared to the control group; Abbreviation: WBC, white blood cell; RBC, red blood cell count; HGB, hemoglobin concentration; HCT, hematocrit; MCV, mean cell volume; MCH, mean cell hemoglobin; MCHC, mean cell hemoglobin concentration; RDW, Red blood Cell distribution width; PLT, platelets; MPV, Mean platelet volume; NEUT, neutrophiles; LYMPH, lymphocytes; MONO, monocytes; EOS, eosinophils; BASO, basophiles; LUC, large unstained cells; RETIC, reticulocyte; PT, Prothrombin time; Fbg, fibrinogen; APTT, activated partial thromboplastin time.

Supplementary table 3. The blood biochemistry analysis of NOG mice treated with hUC-MSCs in the examination of drug withdrawal.

Parameter	Control	Low dose	High dose
Number of animals	12	12	12
ALT (IU/L)	25.69 ± 2.72	23.35 ± 2.95	24.22 ± 6.59
AST (IU/L)	68.59 ± 6.21	67.44 ± 5.36	67.36 ± 16.00
ALP (IU/L)	93.49 ± 24.06	92.50 ± 23.08	93.95 ± 26.88
T.BIL (umol/L)	2.167 ± 0.637	2.167 ± 0.717	2.236 ± 0.716
CK (IU/L)	191.89 ± 112.93	180.63 ± 81.80	198.17 ± 146.10
T.P (g/L)	44.75 ± 1.73	44.32 ± 1.51	45.03 ± 2.00
ALB (g/L)	29.58 ± 1.30	29.16 ± 1.35	29.48 ± 1.48
GLO (g/L)	15.17 ± 0.79	15.16 ± 0.89	15.55 ± 1.03
A/G	1.95 ± 0.11	1.93 ± 0.15	1.90 ± 0.14
GLU (mmol/L)	5.022 ± 1.190	5.266 ± 0.931	6.506 ± 1.082**
BUN (mmol/L)	11.416 ± 0.880	11.588 ± 0.864	12.147 ± 1.382
Crea (umol/L)	34.13 ± 3.31	34.93 ± 2.29	34.97 ± 3.12
T.CHO (mmol/L)	1.583 ± 0.254	1.620 ± 0.298	1.643 ± 0.220
TG (mmol/L)	0.705 ± 0.185	0.580 ± 0.081*	0.614 ± 0.065
K^+ (mmol/L)	4.36 ± 0.34	4.56 ± 0.36	4.46 ± 0.40
Na^+ (mmol/L)	153.6 ± 1.3	154.2 ± 1.4	154.2 ± 0.9
Cl^- (mmol/L)	124.6 ± 4.1	120.1 ± 1.6**	118.9 ± 1.7**
Ca (mmol/L)	2.03 ± 0.08	2.01 ± 0.05	2.03 ± 0.05

Note: The data were expressed as mean ± SD. "*"or"**" indicates a statistically significant difference at p <0.05 and p <0.01 when compared to the control group; Abbreviation: ALT, alanine aminotransferase; AST, aspartate aminotransferase; TP, total protein; ALB, albumin; TBIL, total bilirubin; ALP, alkaline phosphatase; r-GT, r-Glutamyltransferase; GLU, glucose; BUN, Blood urea nitrogen; Crea, Creatinine; CHO, cholesterol; TG, triglyceride; CK, creatine phosphokinase; GLO, Globulin; A/G, albumin/globulin ratio

Supplementary table 4. The Organ weight and coefficient of NOG mice treated with hUC-MSCs in the examination at the end of the recovery period.

Parameter		Male mice			Female mice		
		Control	Low dose	High dose	Control	Low dose	High dose
Number of animals		6	6	6	6	6	6
Body weight (g)		27.0 ± 1.7	25.3 ± 1.4	26.0 ± 1.3	22.0 ± 1.4	21.3 ± 0.9	20.7±2.1
**Brain**	Weight (g)	0.486 ± 0.019	0.476 ± 0.015	0.495 ± 0.014	0.491 ± 0.026	0.499 ± 0.022	0.486±0.017
	Organ coefficient (%)	1.804 ± 0.098	1.888 ± 0.113	1.905 ± 0.097	2.231 ± 0.089	2.347 ± 0.119	2.365±0.175
**Heart**	Weight (g)	0.121 ± 0.013	0.108 ± 0.005	0.114 ± 0.006	0.102 ± 0.011	0.097 ± 0.007	0.096±0.009
	Organ coefficient (%)	0.448 ± 0.044	0.428 ± 0.024	0.440 ± 0.039	0.461 ± 0.038	0.459 ± 0.043	0.466±0.033
	Organ brain coefficient	0.249 ± 0.024	0.227 ± 0.012	0.231 ± 0.012	0.207 ± 0.018	0.195 ± 0.016	0.197±0.016
**Liver**	Weight (g)	1.286 ± 0.134	1.033 ± 0.056**	1.005 ± 0.077**	0.925 ± 0.095	0.825 ± 0.024	0.809±0.108
	Organ coefficient (%)	4.751 ± 0.215	4.085 ± 0.060**	3.860 ± 0.147**	4.203 ± 0.388	3.884 ± 0.155	3.917±0.414
	Organ brain coefficient	2.645 ± 0.242	2.170 ± 0.126**	2.033 ± 0.168**	1.886 ± 0.176	1.657 ± 0.073*	1.662±0.202*
**Spleen**	Weight (g)	0.021 ± 0.002	0.024 ± 0.006	0.019 ± 0.005	0.027 ± 0.005	0.023 ± 0.004	0.023±0.005
	Organ coefficient (%)	0.077 ± 0.006	0.095 ± 0.026	0.073 ± 0.016	0.121 ± 0.021	0.106 ± 0.019	0.109±0.017
	Organ brain coefficient	0.043 ± 0.004	0.050 ± 0.014	0.039 ± 0.010	0.054 ± 0.011	0.045 ± 0.008	0.047±0.010
**Kidney**	Weight (g)	0.357 ± 0.033	0.332 ± 0.036	0.343 ± 0.015	0.244 ± 0.020	0.237 ± 0.017	0.228±0.025
	Organ coefficient (%)	1.321 ± 0.060	1.309 ± 0.088	1.319 ± 0.069	1.106 ± 0.043	1.114 ± 0.086	1.102±0.073
	Organ brain coefficient	0.734 ± 0.055	0.696 ± 0.069	0.693 ± 0.031	0.496 ± 0.018	0.474 ± 0.022	0.468±0.042
**Adrenal gland**	Weight (g)	0.008 ± 0.004	0.007 ± 0.002	0.007 ± 0.002	0.009 ± 0.002	0.008 ± 0.000	0.008±0.002
	Organ coefficient (%)	0.029 ± 0.016	0.029 ± 0.008	0.026 ± 0.006	0.040 ± 0.012	0.037 ± 0.003	0.036±0.009
	Organ brain coefficient	0.016 ± 0.009	0.015 ± 0.005	0.014 ± 0.004	0.018 ± 0.005	0.016 ± 0.001	0.015±0.004
**Testis/ Uterus**	Weight (g)	0.194 ± 0.018	0.187 ± 0.006	0.186 ± 0.009	0.140 ± 0.030	0.156 ± 0.042	0.110±0.031
	Organ coefficient (%)	0.720 ± 0.090	0.739 ± 0.039	0.717 ± 0.050	0.637 ± 0.144	0.729 ± 0.181	0.535±0.158
	Organ brain coefficient	0.398 ± 0.036	0.392 ± 0.016	0.377 ± 0.024	0.286 ± 0.067	0.312 ± 0.080	0.227±0.067
**Epididymis/ Ovary**	Weight (g)	0.094 ± 0.018	0.080 ± 0.018	0.086 ± 0.012	0.024 ± 0.006	0.025 ± 0.004	0.024±0.006
	Organ coefficient (%)	0.348 ± 0.068	0.319 ± 0.077	0.329 ± 0.042	0.111 ± 0.029	0.116 ± 0.019	0.114±0.028
	Organ brain coefficient	0.194 ± 0.040	0.169 ± 0.040	0.173 ± 0.025	0.050 ± 0.014	0.050 ± 0.010	0.049±0.013

Note: The data were expressed as mean ± SD. "*"or"**" indicates a statistically significant difference at p <0.05 and p <0.01 when compared to the control group;
Organ coefficient (%) = g organ weight/g body weight×100%; Organ brain coefficient= g organ weight/g brain weight.

Supplementary table 5. Concentration of human SRY DNA in tissue of NOG mice treated with hUC-MSCs (copies/mg, n=6)

Group	Gender	24 hours after last administration						4w after last administration					
		Skin	Blood	Lung	Kidney	Muscle	Testis/ Uterus	Skin	Blood	Lung	Kidney	muscle	Testis/ Uterus
Low dose	male	B	B	B	B	B	B	B	B	B	B	B	B
		1.99×10^3	B	B	B	B	B	5.69×10^2	B	B	B	B	B
		B	B	B	B	B	B	B	B	B	B	B	B
	female	7.46×10^2	B	B	B	B	B	B	B	$5.61\times10^{4\#}$	B	B	B
		B	B	B	B	B	B	B	B	B	B	B	B
		B	B	B	B	B	B	B	B	B	B	B	B
High dose	male	B	B	B	B	B	B	B	B	B	B	B	B
		1.80×10^5	B	B	B	B	B	1.10×10^3	B	B	B	B	B
		1.10×10^4	B	B	B	B	B	B	B	B	$2.81\times10^{3\#}$	B	B
	female	1.09×10^5	B	B	B	B	B	B	B	B	B	B	B
		4.07×10^3	B	B	B	B	B	B	B	B	B	B	B
		2.13×10^5	B	B	B	B	B	B	B	B	B	$3.95\times10^{4\#}$	B

Note: "B" represents below the lower limit of quantification, which is 100 copies in this methodology/μL. "#" indicates that the data is suspected to be abnormal for the first time, and the data in the table are the results of two retests.

Supplementary table 6. Summary of main reagent information

Reagent Name	Batch Number	Manufacturer	Application
**Diff Timpac Reagent**	61801	Siemens Healthcare Diagnostics	Hematology
**Whole Blood Cell Count Reagent**	75863	Siemens Healthcare Diagnostics	Hematology
**Rinse Sheath Fluid**	67287	Siemens Healthcare Diagnostics	Hematology
**Quality Control (High Value)**	TP223095	Siemens Healthcare Diagnostics	Hematology
**Quality Control (Medium Value)**	TP222095	Siemens Healthcare Diagnostics	Hematology
**Quality Control (Low Value)**	TP221095	Siemens Healthcare Diagnostics	Hematology
**Reticulocyte Staining Reagent**	61749	Siemens Healthcare Diagnostics	Hematology
**EZ Dedicated Cleaning Solution/Agent**	49972	Siemens Healthcare Diagnostics	Hematology
**Cleaning Solution I**	A2069	SYSMAX CORPORATION	Coagulation
**Quality Control I**	564848	Siemens Healthcare Diagnostics Products Gmbh	Coagulation
**Quality Control P**	556730B	Siemens Healthcare Diagnostics Products Gmbh	Coagulation
**Buffer Solution**	569915	Siemens Healthcare Diagnostics Products Gmbh	Coagulation
**Calcium Chloride Solution**	563892A	Siemens Healthcare Diagnostics Products Gmbh	Coagulation
**Activated Partial Thromboplastin Time (APTT) Assay Kit**	557663A	Siemens Healthcare Diagnostics Products Gmbh	Coagulation
**Fibrinogen (Fbg) Assay Kit**	565131	Siemens Healthcare Diagnostics Products Gmbh	Coagulation
**Prothrombin Time (PT) Assay Kit**	568060	Siemens Healthcare Diagnostics Products Gmbh	Coagulation
**Alanine Aminotransferase Assay Kit**	31923/60149796, 32875/60153348	DiaSys Diagnostic Systems GmbH, Germany	Biochemistry
**Aspartate Aminotransferase Assay Kit**	32139/60151655	DiaSys Diagnostic Systems GmbH, Germany	Biochemistry
**Total Protein Liquid Reagent Kit**	231090/50006356	DiaSys Diagnostic Systems GmbH, Germany	Biochemistry
**Albumin Assay Kit**	022082/50006424	DiaSys Diagnostic Systems GmbH, Germany	Biochemistry
**Total Bilirubin Assay Kit**	R1:DK880, R2:DK881	Wako Pure Chemical Industries, Ltd	Biochemistry
**Alkaline Phosphatase Assay Kit**	32489/60153352	DiaSys Diagnostic Systems GmbH, Germany	Biochemistry
**Gamma-Glutamyl Transferase Assay Kit**	31798/60149794	DiaSys Diagnostic Systems GmbH, Germany	Biochemistry
**Glucose Assay Kit**	31232/60148201	DiaSys Diagnostic Systems GmbH, Germany	Biochemistry
**Urea Assay Kit**	31408/60148198	DiaSys Diagnostic Systems GmbH, Germany	Biochemistry
**Creatinine Liquid Reagent Kit**	171046/50006230	DiaSys Diagnostic Systems GmbH, Germany	Biochemistry
**Total Cholesterol Assay Kit**	30908/60148226	DiaSys Diagnostic Systems GmbH, Germany	Biochemistry
**Triglyceride Assay Kit**	31069/60148173	DiaSys Diagnostic Systems GmbH, Germany	Biochemistry
**Creatine Kinase Assay Kit**	31303/60148180	DiaSys Diagnostic Systems GmbH, Germany	Biochemistry
**Calcium Assay Kit**	30706/60148170	DiaSys Diagnostic Systems GmbH, Germany	Biochemistry
**ISE Reference Electrode Solution**	L1607	Hitachi Instruments (Suzhou) Co., Ltd	Biochemistry
**ISE Internal Calibration Solution**	J4478	Hitachi Instruments (Suzhou) Co., Ltd	Biochemistry
**ISE Diluent**	K2295	Hitachi Instruments (Suzhou) Co., Ltd	Biochemistry
**ISE Cleaning Agent**	H1323	Hitachi Instruments (Suzhou) Co., Ltd	Biochemistry
**Normal Value Composite Control**	31257/50273043	DiaSys Diagnostic Systems GmbH, Germany	Biochemistry
**Pathological Value Composite Control**	29696/50275578	DiaSys Diagnostic Systems GmbH, Germany	Biochemistry
**Alkaline Cleaning Solution**	F6748	Hitachi Instruments (Suzhou) Co., Ltd	Biochemistry
**Acid Cleaning Solution**	E2067	Hitachi Instruments (Suzhou) Co., Ltd	Biochemistry
**Antibacterial Phosphorus-Free Cleaning Agent**	C1683, C1705	Hitachi Instruments (Suzhou) Co., Ltd	Biochemistry

Supplementary table 7. Hematological test items

Indicator Name	Abbreviation	Unit	Detection Method	Detection Instrument
**Red Blood Cell Count**	RBC	10^6/μL	Laser Scattering	ADVIA2120
**White Blood Cell Count**	WBC	10^3/μL	Laser Scattering	ADVIA2120
**Hemoglobin**	HGB	g/L	Cyanmethemoglobin Colorimetry	ADVIA2120
**Platelet Count**	PLT	10^3/μL	Two-Dimensional Laser Scattering	ADVIA2120
**Hematocrit**	HCT	%	HCT=(MCV×RBC)÷10	ADVIA2120
**Mean Corpuscular Volume**	MCV	fL	Laser Scattering	ADVIA2120
**Mean Corpuscular Hemoglobin**	MCH	pg	MCH=HGB÷RBC	ADVIA2120
**Mean Corpuscular Hemoglobin Concentration**	MCHC	g/L	MCHC=HGB÷(MCV×RBC)×1000	ADVIA2120
**Mean Platelet Volume**	MPV	fL	Two-Dimensional Laser Scattering	ADVIA2120
**Red Cell Distribution Width**	RDW	%	Laser Scattering	ADVIA2120
**Neutrophil Count**	#NEUT	10^3/μL	#NEUT=(%NEUT×WBC)÷100	ADVIA2120
**Neutrophil Percentage**	%NEUT	%	Peroxidase Staining	ADVIA2120
**Lymphocyte Count**	#LYMPH	10^3/μL	#LYMPH=(%LYMPH×WBC)÷100	ADVIA2120
**Lymphocyte Percentage**	%LYMPH	%	Peroxidase Staining	ADVIA2120
**Monocyte Count**	#MONO	10^3/μL	#MONO=(%MONO×WBC)÷100	ADVIA2120
**Monocyte Percentage**	%MONO	%	Peroxidase Staining	ADVIA2120
**Eosinophil Count**	#EOS	10^3/μL	#EOS=(%EOS×WBC)÷100	ADVIA2120
**Eosinophil Percentage**	%EOS	%	Peroxidase Staining	ADVIA2120
**Basophil Count**	#BASO	10^3/μL	#BASO=(%BASO×WBC)÷100	ADVIA2120
**Basophil Percentage**	%BASO	%	Peroxidase Staining	ADVIA2120
**Large Unstained Cell Count**	#LUC	10^3/μL	#LUC=(%LUC×WBC)÷100	ADVIA2120
**Large Unstained Cell Percentage**	%LUC	%	Peroxidase Staining	ADVIA2120
**Reticulocyte Count**	#RETIC	10^9/L	Erythrocyte Spheroidization and RNA Staining	ADVIA2120
**Reticulocyte Percentage**	%RETIC	%	%RETIC=(#RETIC÷RBC)÷10	ADVIA2120
**Prothrombin Time**	PT	s	Agglutination Method	CA-1500
**Activated Partial Thromboplastin Time**	APTT	s	Agglutination Method	CA-1500
**Fibrinogen**	Fbg	g/L	Agglutination Method	CA-1500

Supplementary table 8. Serum biochemical test items

Indicator Name	Abbreviation	Unit	Detection Method	Detection Instrument
**Aspartate Aminotransferase**	AST	IU/L	UV Continuous Monitoring Method	Hitachi 7180
**Alanine Aminotransferase**	ALT	IU/L	UV Continuous Monitoring Method	Hitachi 7180
**Alkaline Phosphatase**	ALP	IU/L	Rate Method	Hitachi 7180
**Creatine Phosphokinase**	CK	IU/L	IFCC Continuous Monitoring Method	Hitachi 7180
**Blood Urea Nitrogen**	BUN	mmol/L	Glutamate Dehydrogenase Method	Hitachi 7180
**Creatinine**	Crea	μmol/L	Picric Acid Method	Hitachi 7180
**Total Protein**	T.P	g/L	Biuret Method	Hitachi 7180
**Albumin**	ALB	g/L	Bromocresol Green Method	Hitachi 7180
**Globulin**	GLO	g/L	GLO=T.P-ALB	Hitachi 7180
**Albumin/Globulin Ratio**	A/G	/	A/G=ALB÷(T.P-ALB)	Hitachi 7180
**Blood Glucose**	GLU	mmol/L	Hexokinase Method	Hitachi 7180
**Total Bilirubin**	T.BIL	μmol/L	Vanadate Oxidation Method	Hitachi 7180
**Total Cholesterol**	T.CHO	mmol/L	Enzyme Reagent Method	Hitachi 7180
**Triglyceride**	TG	mmol/L	Enzyme Reagent Method	Hitachi 7180
**Calcium**	Ca	mmol/L	Arsenazo III Method	Hitachi 7180
**Potassium Ion**	K+	mmol/L	Electrode Method	Hitachi 7180
**Sodium Ion**	Na+	mmol/L	Electrode Method	Hitachi 7180
**Chloride Ion**	Cl-	mmol/L	Electrode Method	Hitachi 7180
